# Direct selection and phage display of a Gram-positive secretome

**DOI:** 10.1186/gb-2007-8-12-r266

**Published:** 2007-12-13

**Authors:** Dragana Jankovic, Michael A Collett, Mark W Lubbers, Jasna Rakonjac

**Affiliations:** 1 Institute of Molecular Biosciences, Massey University, Palmerston North, New Zealand; 2 Fonterra Research Centre, Palmerston North, New Zealand; 3 Fonterra, Mount Waverley, VIC 3149, Australia

## Abstract

Surface, secreted and transmembrane protein-encoding open reading frames, collectively the secretome, can be identified in bacterial genome sequences using bioinformatics. However, functional analysis of translated secretomes is possible only if many secretome proteins are expressed and purified individually. We have now developed and applied a phage display system for direct selection, identification, expression and purification of bacterial secretome proteins.

## Abstract 2

A phage display system for direct selection, identification, expression and purification of bacterial secretome proteins has been developed.

## Background

The secretome comprises a wide range of proteins that mediate interactions with the environment, such as receptors, adhesins, transporters, complex cell surface structures such as pili, secreted enzymes, toxins and virulence factors. In bacteria that colonize the human organism, secreted proteins mediate attachment to the host, destruction of the host tissue or interference with the immune response [1-3]. In pathogenic bacteria, variation of a surface protein between strains of a species can indicate its role in evading the immune response [4-7]; conversely, conserved surface proteins that are capable of inducing a protective immune response are sought for as vaccine candidates [8]. 'Mining' the secretome is essential for a range of applications; from identifying potentially useful enzymes, to understanding virulence [1-3,8-13].

Secretome proteins contain membrane targeting sequences - signal sequences and transmembrane α-helices. There are several types of signal sequences: the 'classic' or type I signal sequence, the twin arginine translocon (Tat) signal sequence, the lipoprotein or type II signal sequence, and the prepilin-like or type IV signal sequence. A secretome can be deduced from a completely sequenced genome by using a range of available algorithms that can identify signal sequences and transmembrane α-helices, for example, SignalP 3.0, TMHMM 2.0, LipoPred, or PSORT [14-19]. However, obtaining complete genome sequences of multiple bacterial strains in order to identify their secretomes is inefficient because the secretome is a minor portion of the genome, typically comprising only 10-30% of the total number of the open reading frames (ORFs) [10]. An approach in which the secretome sequences were specifically selected prior to sequence analysis would dramatically increase the efficiency of identifying secretome proteins, compared to the conventional shotgun sequencing approach [20,21].

Purely bioinformatic analysis is not only inefficient for secretome protein identification, but also does not provide the means for direct functional characterization of identified proteins. In the post-bioinformatics phase of genome research, candidate ORFs are usually chosen based on a sequence motif or homology to a protein of known function, and then are either mutated by reverse genetics, or the protein products are expressed, purified and directly characterized. Both of these approaches are very demanding. The former requires that a reverse genetics method exists for the organism of interest; the latter is complicated by the fact that the secretome proteins are notoriously hard to express and purify [22].

Phage display technology offers a very efficient way to purify and characterize proteins by displaying them on the surface of the bacteriophage virion [23,24]. Filamentous phage virions that display foreign proteins can also act as purification tags, being very simply purified from culture supernatants by precipitation with polyethylene glycol (PEG). Display is achieved by translational fusion of a protein or library of proteins of interest to any of the five virion proteins, although the pIII and pVIII proteins are used most frequently [25,26]. Filamentous phage virion proteins are themselves secretome proteins, translocated from cytoplasm via the Sec-dependent pathway and anchored in the cytoplasmic membrane prior to assembly into the virion [27,28]. Therefore, the secretome proteins to be displayed would be targeted to, and folded in, the cellular compartment in which they normally reside. Phage display combinatorial libraries are widely used to identify rare protein variants that bind to complex ligands of interest; the most complex example reported being an *in vivo *screen for peptides that bind endothelial surfaces of the capillaries in an organ-specific fashion [29]. Furthermore, phage display screening methods for selection and *in vitro *evolution of enzymes have been developed and used successfully [30].

Phage protein pIII is the most frequently used display platform; it contains a signal sequence, which is the hallmark of the majority of the secretome proteins. A signal sequence is necessary for correct targeting of pIII to the inner membrane and incorporation into the virion [31]. Moreover, assembly of pIII into the virion is required to complete the phage assembly. When pIII is absent, virions either stay associated with the host cells as long filaments composed of multiple sequentially packaged genomes, or are broken off by mechanical shearing. pIII is required for formation of the stabilizing cap structure at the terminus of the virion; hence, the broken-off pIII-deficient virions are structurally unstable and are easily disassembled by sarcosyl, to which the pIII-containing virions are resistant [32,33]. We exploited this requirement to create a direct selection scheme for cloning and display of the secretome proteins and applied it to identifying the secretome of the probiotic bacterium *Lactobacillus rhamnosus *HN001 [34-36].

Probiotic bacteria have been shown previously to induce beneficial health effects, but the molecular mechanism and the proteins involved are still being elucidated [37,38]. Some evidence suggests that probiotic bacteria can competitively adhere to intestinal mucus and displace pathogens [39-42]. The adherence of probiotic bacteria to human intestinal mucus and cells appears to be mediated, at least in part, by secretome proteins [13,43-47]. A large body of work on pathogenic bacteria has demonstrated a key role for secretome proteins in more complex interactions with the host, such as modulation of immune response; it is thus expected that surface and secreted proteins also play a major role in complex interactions between probiotic bacteria and the human organism. We demonstrated the efficiency of our secretome selection method by identifying and displaying 89 surface and secreted proteins, seven of which were unique to *L. rhamnosus *HN001.

## Results

### Construction of the secretome-selective phage display system

**Figure 1:**
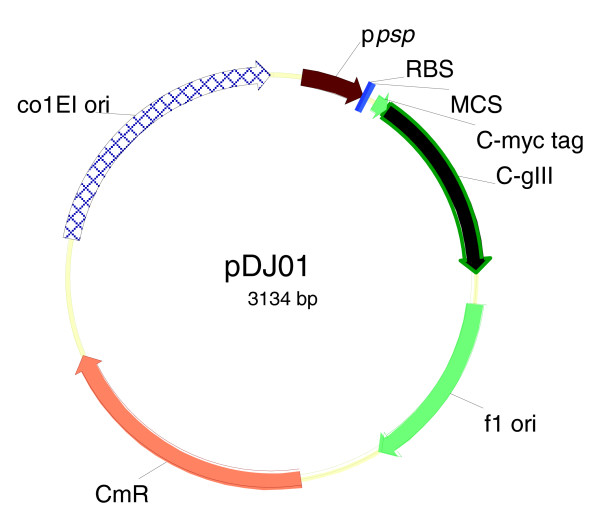
Phage display vector for selective secretome display. C-gIII, carboxy-terminal domain of *gIII*; Cm^R^, chloramphenicol resistance cassette; colE1 ori, the colE1 plasmid origin of replication; p*psp*, phage shock protein promoter; MCS, multiple cloning site; RBS, ribosomal binding site; C-myc, a common peptide tag followed by a single *amber *stop codon; f1 ori, the f1 phage origin of replication for generation of ssDNA for packaging into the phagemid particles. The stop codon is read as glutamic acid in the host strain TG1 (*supE*) used in the library construction and screening, allowing read-through into the in-frame *gIII*-coding sequence and display on the phage. Expression of the soluble secretome proteins tagged with the C-myc peptide tag (without pIII moiety) can be achieved by using a suppressor-negative *E. coli *host strain.

A typical phage display system consists of two components: phagemid vector and a helper phage [26]. The phagemid vectors most commonly encode the carboxy-terminal domain of pIII, preceded by a signal sequence. Inserts are placed between the signal sequence and mature portion of pIII. If an insert is translationally in-frame with both the signal sequence and the mature portion of pIII, then the encoded protein will be displayed on the surface of the phage. The first step in development of the secretome selection and display system was construction of a new phagemid vector, pDJ01, containing a pIII C-domain cloning cassette from which the signal sequence was deleted (Figure 1). The helper phage component of a phage display system is normally used to provide the f1 replication protein pII that mediates the rolling circle replication of the phagemid vector from the f1 origin, resulting in a single-stranded DNA (ssDNA) genome that is packaged into the virion [48]. The helper phage also provides other phage-encoded proteins essential for packaging of the phagemid ssDNA into the virion, to form phagemid or transducing particles. However, the helper phage that we used had the entire coding sequence for pIII(*gIII*) removed [49]. Hence, the only pIII protein expressed in our system was the phagemid vector-encoded pIII that lacked a signal sequence. To test whether pIII without signal sequence would lead to production of incomplete (defective) phagemid particles, cells containing pDJ01 were infected with the Δ*gIII *helper phage VCSM13d3 [49] to generate phagemid particles. Sarcosyl treatment of these phagemid particles resulted in their disassembly and release of the phagemid ssDNA (not shown), confirming that these particles were indeed defective.

### pIII fusion to Gram-positive signal sequence completes the phage assembly and displays functional Gram-positive secretome protein

**Figure 2:**
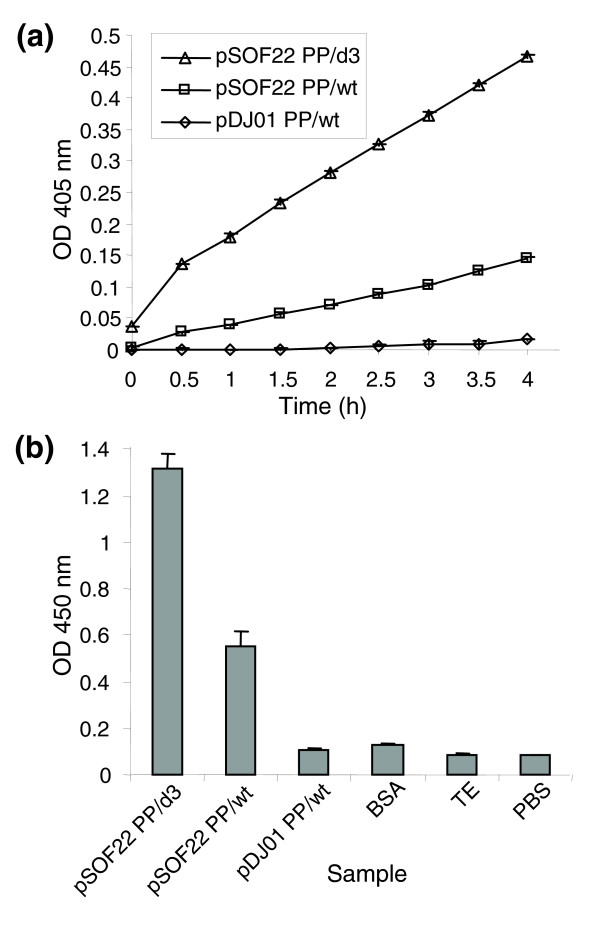
Biological activities of the serum opacity factor targeted to the phage by a Gram-positive signal sequence. **(a) **The serum opacity activity of the pSOF22 phagemid particles displaying the SOF22. A total of 10^11 ^phagemid particles were used per 200 μl assay. **(b) **Binding of the SOF22-displaying phagemid particles to human fibronectin detected by phage ELISA. A total of 10^8 ^phagemid particles were used per assay, each carried out in a well of a 96-well plate. Samples: pSOF22 PP/d3 and pSOF22 PP/wt, phagemid particles displaying the SOF from *S. pyogenes *M22, generated using VCSM13d3 and VCSM13 helper phage, respectively; pDJ01 PP/wt, the vector phagemid particles, generated using the VCSM13 helper phage. BSA, TE, PBS, and BSA are buffer controls. Each data point is an average of three replicas; error bars represent standard deviation.

The hallmark of a signal sequence is a hydrophobic α-helix of at least 15 amino acid residues in length at the amino terminus of the protein. In bacteria, this helix is preceded by a few residues, predominantly positively charged, and is followed by either electroneutral or negatively charged residues [50]. pIII has an 18-residue signal sequence, which is normally processed by Gram-negative secretion machinery in the *Escherichia coli *host. However, Gram-positive signal sequences are significantly longer than those of Gram-negative bacteria [51] so it was not clear whether they would be processed with sufficient efficiency in *E. coli *to allow production of functional pIII. We tested this by inserting into pDJ01, in-frame with gIII, a surface protein from a Gram-positive bacterium (the serum opacity factor of *Streptococcus pyogenes*, M-type 22 (SOF22)) [52]. The SOF22 portion of the protein fusion was 963 amino acid residues in length (including the signal sequence), and it lacked the cell wall and membrane anchor sequences located at the very carboxyl terminus of the protein. Importantly, the signal sequence of SOF22 is 40 residues in length, approximately twice as long as that of pIII. Therefore, this is an example of a typical Gram-positive bacterial secretome protein that might be found, for example, in the intestinal microflora. Phagemid particles of the pDJ01::SOF22 clone (named pSOF22) were assembled using the pIII-deficient Δ*gIII *helper phage VCSM13d3. These phagemid particles were resistant to sarcosyl (not shown). Therefore, the cap structure was formed, implying that SOF22-pIII fusion was correctly targeted to the virion and that the Gram-positive signal sequence of the SOF22 protein was functional in the *E. coli *host. Furthermore, purified phagemid particles were examined for two biological activities of the displayed SOF22: opacification of the mammalian sera and binding to human fibronectin (Figure 2). SOF22 was displayed by using either the *gIII*-deleted helper phage VCSM13d3 as described above, or *gIII*-positive helper phage, VCSM13. The former resulted in occupancy of all pIII positions in the phagemid particles with the SOF22-pIII fusions, and the latter in a mixture of the SOF22-pIII fusion and the wild-type pIII from the *gIII*-positive helper phage VSCM13. Purified particles demonstrated both opacification and fibronectin binding activities. Consistent with the expected higher copy number of SOF22-pIII fusions when VCSM13d3 is used as the helper phage, both serum opacity and fibronectin-binding activities were greater in the phagemid particles produced by infection with the *gIII*-deleted helper phage VCSM13d3 (Figure 2). Retention of biological activity of SOF22 suggests that large proteins of Gram-positive bacteria can be displayed and properly folded in this system, despite containing a signal sequence that is much longer than the native signal sequence used by pIII.

### Selection of the *Lactobacillus rhamnosus *HN001 secretome

A mock experiment was carried out to establish a selection protocol and estimate the efficiency of selective enrichment achieved for secretome clones. Defective pDJ01 phagemid particles were mixed with complete pSOF22 phagemid particles at a ratio of 100 to 1, respectively (both types of phagemid particles were generated using the Δ*gIII *helper phage VCSM13d3 as described in previous sections). A selection protocol was then developed to remove the signal sequence-negative pDJ01 (empty vector) from the mixture while preserving the signal sequence-positive phagemid pSOF22. Sarcosyl was first added to the mixture to disassemble the defective pDJ01 phagemid particles; DNase I was then used to remove the pDJ01 ssDNA released from disassembled phagemid particles, followed by inactivation of DNase I by EDTA. The remaining sarcosyl-resistant phagemid particles were then disassembled by heating in SDS and the released ssDNA was purified and transformed into a new *E. coli *host. Analysis of *E. coli *transformed with purified ssDNA showed that the secretome protein-encoding clone pSOF22 was enriched 800-fold over the vector pDJ01 (from 1:100 to 8:1), indicating that the newly developed selection protocol was highly efficient in this mock selection experiment. The background of the empty vector remaining after the selection could not be further reduced by increasing the amount or the length of incubation with DNase I.

**Figure 3:**
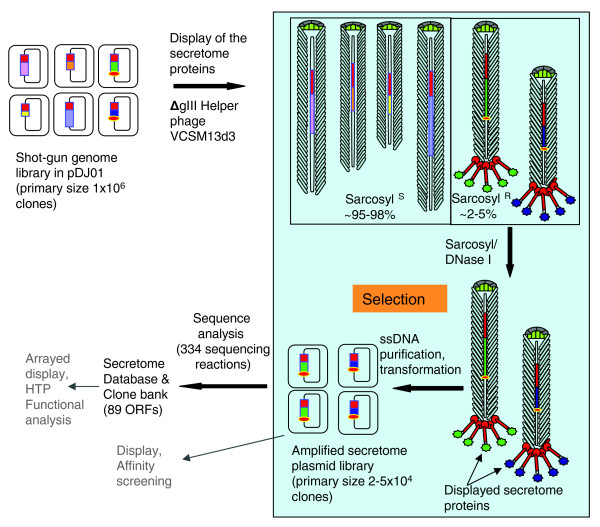
The secretome selection diagram. The key selection steps are boxed. Rounded squares represent *E. coli *cells and rounded rectangles represent recombinant phagemids replicating as plasmids inside the cells. pIII is shown as a red rectangle on the plasmid backbone. Inserts are represented as rectangles of various colors and lengths. Small orange ovals represent the signal sequences. The pipe-cleaner-like shapes represent phagemid particles obtained after infection of the library with the helper phage VCSM13d3. The elongated rectangles along the axes represent packaged DNA of the library clones. The top ends of the phagemid particles contain pVII and pIX proteins. The bottom ends of the phagemid particles are either open (signal sequence-negative clones) or capped by protein-pIII fusions (signal sequence-positive clones; popsicle shapes). Sarcosyl^S^, phagemid particles sensitive to sarcosyl; sarcosyl^R^, the secretome protein-displaying phagemid particles, resistant to sarcosyl. Numbers in brackets refer to data obtained in the *L. rhamnosus *HN001 secretome selection experiment in this work. Steps denoted in grey indicate downstream applications of the secretome library.

To examine the efficiency of selection of a secretome phage display library, the above method was used to identify the secretome of the Gram-positive probiotic bacterium *L. rhamnosus *HN001 (Figure 3). A small-insert shotgun genomic library was created in the pDJ01 vector. The insert size ranged from 0.3 to 4 Kbp and the primary size of the library was 10^6 ^clones. The library was first amplified using the plasmid origin of replication (in the absence of a helper phage). In the next step, the amplified library was mass-infected with the Δ*gIII *helper phage VCSM13d3 [49] to initiate replication of the phagemid from the f1 origin and packaging into the phagemid particles. Based on the preliminary experiment described in the previous paragraph, inserts encoding the signal sequence-containing proteins in-frame with pIII were expected to restore its function and allow assembly of the terminal cap of the virions, rendering them resistant to sarcosyl. These resistant phagemid particles were expected to display the pIII-secretome protein fusions on the surface and contain the corresponding DNA sequence inside the phagemid particle. In contrast, defective phagemid particles that lack an insert encoding a signal sequence-containing protein that is translationally fused to *gIII *were expected to be disassembled in the presence of sarcosyl. Thus, sarcosyl treatment would release the recombinant phagemid ssDNA encapsidated in the defective phagemid particles; the released DNA would then be digested by DNase I and eliminated in the selection step.

**Figure 4:**
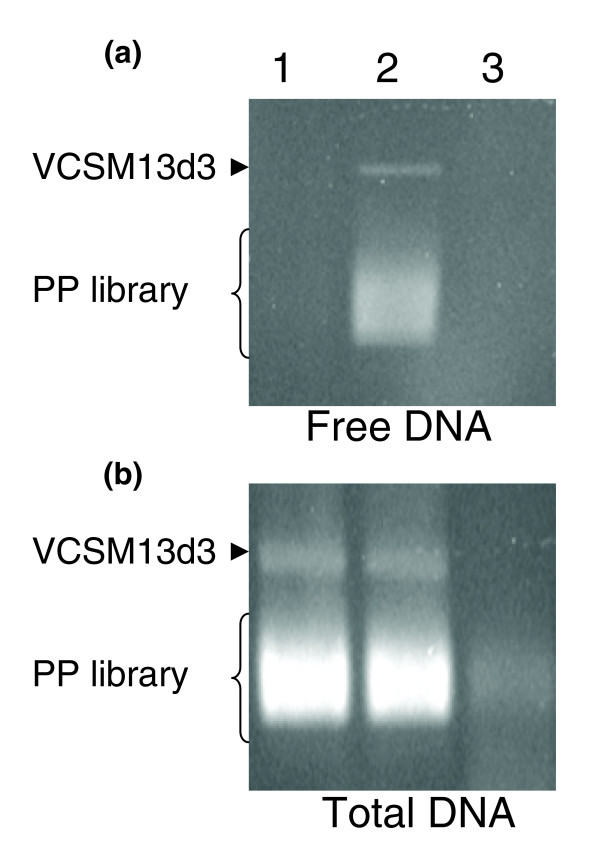
Demonstration of the sarcosyl resistance selection step. **(a) **Free phagemid DNA (samples were loaded directly on a 0.8% agarose gel); **(b) **total DNA, the sum of the free DNA and DNA encapsulated in the phagemid particles (samples were heated at 70°C in 1.2% SDS for 10 minutes before loading, to disassemble the sarcosyl-resistant phagemid particles). Lanes: 1, library phagemid particles (PP) before incubation with sarcosyl; 2, after incubation with sarcosyl; 3, after incubation with sarcosyl and DNase I, followed by inactivation of DNase I).

After infection with VCSM13d3 helper phage, the library was incubated on a solid medium to minimize growth competition among the library clones. Phagemid particles released from the infected library were collected and purified by PEG precipitation (as described in Materials and methods). Sarcosyl-induced release of phagemid DNA was monitored by agarose gel electrophoresis and staining with ethidium bromide (Figure 4a, compare lanes 1 and 2). The sarcosyl-released ssDNA was eliminated by DNase I (Figure 4a, lane 3). The total DNA in the virions (both encapsulated and free) was detected by disassembling all virions, both defective and pIII-containing, with SDS at 70°C, prior to electrophoresis. The electrophoresis of SDS-disassembled virions detected a weak signal in the post-DNase treatment samples compared to the signal from the sarcosyl-sensitive phagemid particles. This indicated that, as expected, the majority of the inserts were packaged into sarcosyl-sensitive phagemid most likely because they lacked in-frame signal sequence fusions to the vector pIII. A minority of inserts was packaged into sarcosyl-resistant virions and, therefore, probably contained in-frame signal sequence fusions with the vector pIII (Figure 4b, lane 3). Densitometric analysis indicated that approximately 2-5% of the total phagemid particles were sarcosyl-resistant. This matches the expected frequency of 3.3% or 1/30 [~1/5 (frequency of secretome-encoding ORFs) × 1/2 (probability of correct insert orientation) × 1/3 (probability of the correct frame fusion of the inserts to pIII)].

### Efficiency of the secretome library selection

DNA from the sarcosyl-resistant phagemid particles was purified and transformed into a new *E. coli *host. In the absence of a helper phage, transformed recombinant phagemids replicate from the plasmid origin of replication to form double-stranded DNA in the *E. coli *host. The resulting double-stranded recombinant phagemid DNA was purified from individual colonies and the library inserts were subjected to sequence analysis. Initially 192 inserts were sequenced and a few 'promiscuous' recombinant phagemids that appeared in more than 5 independent transformants were identified. To avoid repeated sequencing of these inserts, a mixture of probes derived from them was used to screen a further 299 transformants by dot-blot hybridization. This revealed 157 recombinant phagemids containing promiscuous inserts and 142 non-promiscuous phagemids that were analyzed by sequencing. In total, 491 library inserts were characterized: 334 by sequencing and 157 by hybridization only. For the inserts that were sequenced, one sequencing reaction was done using a reverse primer complementary to the *gIII *sequence of the vector. If the 5' end of the secretome ORF was not reached, an additional sequencing reaction was done using the forward primer complementary to the vector sequence upstream of the insert. The insert sequences whose translated products in-frame with pIII were longer than 24 residues were analyzed by SignalP 3.0, TMHMM 2.0 and LipPred [14,53] to predict whether they contained any membrane-targeting signals. This revealed that 411 (84%) of the 491 inserts analyzed (sequenced or screened by dot-blot hybridization) contained 87 distinct ORFs predicted to encode secretome proteins in-frame with pIII. Of the remaining 80 non-secretome inserts, 52 contained inserts encoding very short peptides in-frame with pIII (< 24 residues), 12 were empty vector and the remaining 16 inserts encoded peptides longer than 24 residues in-frame with pIII, but these peptides lacked typical membrane-targeting sequences. When infected with Δ*gIII *helper phage VCSM13d3, 14 of these 16 recombinant phagemids failed to assemble sarcosyl-resistant phagemid particles. However, the remaining two recombinant phagemids with no detectable in-frame membrane targeting signals were still able to generate the sarcosyl-resistant phagemid particles that contained the predicted ORF-pIII fusions (data not shown). This strongly suggests that the two inserts contained concealed or perhaps Sec-independent sequences that allowed proper targeting of pIII in the inner membrane of *E. coli*. These two inserts contained ORFs encoding putative folding enzyme disulfide isomerase (*lrh88*) and Cof-like hydrolase (*lrh89*). The subcellular location of homologues of these two enzymes has been reported as in either the periplasm or the cytoplasm [54-58]. However, the two ORFs that we have selected did not encode the signal sequences normally present in the family members that are targeted to the membrane. Hence, the mechanism of the targeting of these two fusions remains unresolved and could potentially involve a conserved Sec/Tat-independent mechanism. In summary, most of the non-secretome clones (50 out of 52) were most likely obtained due to the incomplete digestion of released ssDNA by DNase I in the selection step, rather than mistargeting of the pIII fusions.

Of the 87 ORFs that encoded proteins with predicted membrane-targeting sequences, 46 contained a type I signal sequence (Table 1; see Additional file 1 for the complete list of targeting sequences and secretome ORF annotation). Thirteen ORFs encoded proteins with a predicted lipoprotein signal sequence and 18 with a predicted amino-terminal membrane anchor. Ten ORFs encoded proteins with predicted internal transmembrane α-helices; of those, three have a predicted single transmembrane α-helix and seven have predicted multiple transmembrane α-helices. Notably, 43 out of 89 putative membrane-targeting sequences that have been selected by our method are not type I signal sequences. Given that the type I pIII signal sequence must be cleaved off by the *E. coli *signal peptidase in order to release its amino terminus from the membrane, the non-type I membrane-targeting sequences found in our pIII fusions appear to have been successfully processed in the *E. coli *periplasm, either by the signal peptidase or by some other membrane or periplasmic protease [59]. No inserts containing predicted Tat signal sequences were identified by the available software or manual inspection [60]. This is consistent with other *Lactobacillus *species, none of which contain the Tat translocon [61-67].

**Table 1:** Types of *L. rhamnosus *HN001 membrane-targeting sequences and distribution

Membrane-targeting signal in the insert	Bitopic or extracellular proteins	Polytopic integral membrane proteins	Total
Type I signal sequence	46*		46
Lipoprotein signal sequence	13		13
Amino-terminal transmembrane helix	12	6	18
Internal transmembrane helix	2	1	3
Multiple transmembrane helices		7	7
No recognizable membrane-targeting signals	2		2
Total	75	14	89

*Numbers refer to the number of secretome ORFs predicted to contain a particular type of membrane-targeting signal.

**Figure 5:**
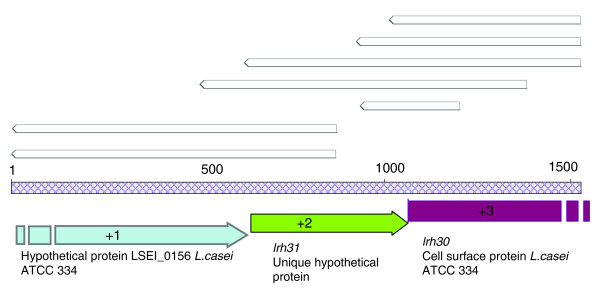
Contig corresponding to ORFs in a secretome protein operon. Top, white arrow-shaped boxes, individual sequence reads, each from a different transformant. Middle, grey cross-hatched box, the contig. Bottom, predicted ORFs with indicated frame and annotation. The first and the third ORFs are partial. The first ORF was not assigned an *lrh *number because it was not directly selected in our screen as a secretome protein.

The enrichment of the secretome insert-containing recombinant phagemids was approximately 210-fold (from approximately 1:40 to 5.26:1), suggesting that the stringency of selection was high and that most recombinant phagemids containing non-secretome inserts were eliminated. Of the 89 secretome ORFs identified, over half (49) were present mulitple times (between 2 and 5) as distinct recombinant phagemids with different points of fusion to pIII. Analysis of DNA sequence contigs, obtained by assembly of individual sequence reads, indicated that some of these ORFs were organized into operons encoding secretome proteins. For example, one contig encoded two secretome ORFs (*lrh31 *and *lrh30*) that were located adjacent to each other within a larger operon (Figure 5). A clone bank and a database of the *L. rhamnosus *HN001 secretome clones were generated from the sequence data and were used for bioinformatic characterization of the secretome.

### Annotation of *L. rhamnosus *secretome proteins

Of the 89 identified ORFs, functions were predicted for 48, comprising 7 functional categories (Table 2). The largest functional category comprised 22 ORFs encoding putative transport proteins, with 13 of these having similarity to extracellular substrate binding domains of ABC transporters and each containing a predicted amino-terminal lipoprotein signal sequence [12]. The remaining nine ORFs in the transport protein category were predicted to encode polytopic transmembrane proteins, with one or more internal transmembrane α-helices.

**Table 2:** Annotation of *L. rhamnosus *HN001 secretome ORFs

Category	Number of ORFs
Enzymes	20
Signal transduction components	1
Transport	22
Host/microbial interactions	3
Conserved, unknown function	28
Unique hypothetical proteins	7
Miscellaneous functions	2
Unclassified*	6
Total	89

*Short fragments of ORFs (encoding 27-57 amino acids) were fused to *gIII*; no hits above the threshold (e^-10^) were detected using BlastP with automatic detection of short sequences. These ORFs were not classified as unique because the short length has prevented identifying potentially significant hits.

ORFs encoding predicted enzymes were the second-largest category. This diverse class included predicted proteases, hydrolases, enzymes involved in cell wall turnover, autolysins and a dithiol-disulfide isomerase (Table 2). One ORF, *lrh15*, had similarity to a sensor histidine protein kinase of *Lactobacillus casei *for which the signal/substrate specificity has not yet been determined.

Several ORFs had significant sequence similarity with known surface proteins. For example, ORF *lrh51 *encodes a predicted protein that is similar to a predicted LPxTG-anchored adhesion exoprotein from *L. casei *ATCC 334. The protein family to which Lrh51 belongs appears to be unique to the *L. casei-Pediococcus *group [68] and may play a role in adaptation to the common environment(s) of these two groups. Another ORF, *lrh35*, encodes a predicted protein homologous to a collagen adhesin of *Bacillus clausii *KSM-K16. One ORF, *lrh17*, encodes a predicted protein containing a pilin motif and partial E-box motif, which are motifs present in the major pilin proteins of Gram-positive bacteria [69]. Analysis of the putative full-length *lrh17 *ORF identified in the draft genome sequence of *L. rhamnosus *HN001 revealed the complete E-box and the cell wall sorting signal; therefore, *lrh17 *is likely to encode the major pilin protein of putative *L. rhamnosus *pili. One of the ORFs, *lrh08*, had sequence similarity to conserved hypothetical proteins that are similar to cell wall-anchored proteins, but appeared to be truncated due to a TAG stop codon. This ORF was probably translated through the TAG stop codon and displayed as pIII fusion because the *E. coli *host strain that we have used contains a *supE *mutation that reads the TAG stop codon as glutamic acid.

Database searches did not reveal any sequences similar to seven of the ORFs. Proteins apparently encoded by these ORFs seem to be unique to *L. rhamnosus *HN001 and, therefore, might potentially be involved in strain-specific interactions between this bacterium and its environment that might be associated with its probiotic effects. One of these ORFs, *lrh62*, encodes a putative serine- and alanine-rich extracellular protein. The insert in the recombinant phagemid encodes 807 residues, but the protein encoded by this gene is predicted to be 2,827 amino acids in length and to contain an LPxTG carboxy-terminal cell wall anchoring motif (as deduced from the draft *L. rhamnosus *HN001 genome sequence). The presence of many alanines (965/2,827) and serines (496/2,827) and the overall protein size is reminiscent of large serine-rich repeat-containing adhesins of Lactobacilli and Streptococci [66]. However, these adhesins typically contain hundreds of copies of a short and highly conserved serine/alanine-rich motif, whereas the alanine and serine residues of ORF *lrh62*, although highly repetitive throughout the protein due to their large numbers, do not appear to form conserved and regularly repeating motifs that could be revealed by self-alignment matrix analysis.

## Discussion

We describe a new system for direct selection, expression and display of the secretome, based on the requirement of a signal sequence for assembly of sarcosyl-resistant filamentous phage virions. While a phage display system for cloning secretome proteins has been previously reported [70] it is not efficient for enrichment and display of Gram-positive secretome proteins. That system uses *gIII*-positive helper phage and the signal sequence-encoding inserts are affinity-enriched based on the presence of a vector-encoded affinity tag incorporated into the fusion. Therefore, the secretome-pIII fusions must successfully compete with the helper phage-derived wild-type pIII for incorporation into the virion. The efficiency of that system for recovery of Gram-positive secretome proteins is poor, with two successive rounds of affinity selection and amplification resulting in only 52 secretome ORFs from a library of the primary size of 10^7 ^clones [71]. Our system resulted in 89 secretome ORFs from a library of only 10^6 ^clones, hence performing about 20-fold more efficiently than the previously reported enrichment method. The much lower efficiency of the previously published system could be explained by low efficiency of processing the Gram-positive signal sequences compared to the wild-type pIII signal sequence. As a consequence, a significant number of secretome proteins would be out-competed by the native pIII of the helper phage and would fail to be incorporated into the phagemid particles, preventing their affinity selection. The much higher efficiency of our method is due to direct selection for the release of the correctly assembled phagemid particles. Wild-type pIII is not present in the system; hence, the recombinant fusions cannot be outcompeted by native pIII. Furthermore, the previously reported system [70] uses a vector with a very strong constitutive promoter that likely confers toxic effects to the host *E. coli*, known to be sensitive to overexpression of pIII fusions [72,73]. As a result, many clones that impair growth of the host *E. coli *and phage assembly would have been lost. Our display system has the advantage of using the very tightly regulated *psp *promoter. This promoter is induced by infection of individual cells with helper phage; it does not require addition of inducer compound or washing away of an inhibitor [74] and has also been shown to improve display of pIII fusion proteins that are toxic to *E. coli *when overexpressed [75]. This promoter allows the expression of ORFs that do not contain their own transcriptional signals, such as those located within operons and distal to the promoter in genomic libraries, as well as expression of coding sequences in cDNA libraries.

Bioinformatic elucidation of the meta-secretome of complex microbial communities, such as those that colonize the human gastrointestinal tract, is impractical with current sequencing technologies because of the poor coverage of the metagenome gene pool, even in large-scale projects [20,21]. Our system's high efficiency secretome selection would allow selective cloning, sequencing, and functional analyses of surface and secreted proteins on a metagenomic scale, where the limiting factor is the initial size of the library [20,76]. Based on the estimated size of the *L. rhamnosus *genome (approximately 3 Mb; W Kelly, personal communication) and the percentage of the secretome clones in Lactobacilli [13], the coverage of the secretome that we achieved is likely to be about 44%. To provide similar coverage of a metagenome with about 100 dominant species, our method would require a primary library size of approximately 10^8 ^and approximately 50,000 sequencing reactions, both of which are easily achievable by standard techniques. Furthermore, Gram-positive *Firmicutes *(*Clostridiales*, *Bacilliales *and *Lactobacilliales*) and *Actinobacteria *(*Actinomycetales *and *Bifidobacteriales*) are dominant groups of bacteria in the human gut microbial community [20,76]. Hence, the highly efficient selection of Gram-positive bacterial secretome ORFs achieved by our direct selection method is crucial to avoid the secretome library being dominated by Gram-negative secretome proteins [77]. Bioinformatic studies of archaeal signal sequences suggest that they closely resemble those of bacteria. It is therefore expected that archaeal signal sequences would be selected using this method [78,79]. In contrast, proteins exported via Tat and Sec-independent translocation pathways of Gram-negative bacteria (type I and III secretion systems) would presumably be absent due to the fundamentally different mechanisms of translocation through the bacterial envelope [51,80,81].

Several reporter fusion systems and cell surface display screening methods have been used to identify secretome proteins and even to systematically analyze the topology of membrane proteins [43,82-86]. However, a distinct advantage of phage display is that the protein is automatically purified by association with the virion, simplifying functional characterization. We have shown that phagemid particles assembled by incorporation of the 963-residue surface protein SOF of the Gram-positive bacterium *S. pyogenes*, targeted by its intrinsic signal sequence, demonstrate two biological activities of this protein corresponding to two independently folding domains. Hence, display and folding of this protein in the context of the phage virion must be reasonably efficient and accurate. Therefore, proteins with an activity of interest could be identified by arraying the secretome clone bank and using high-throughput activity screening. Alternatively, the 'raw' secretome phage display library pool, obtained after the selection step, could be screened for activities of interest by well-established phage display library screening protocols. Applied to microbial communities at a metagenomic scale, these methods would allow functional analysis of proteins from yet uncultivated bacteria.

Bacteria of the *Lactobacillus *genus are found in diverse environments. Some are indigenous to various compartments of the gastrointestinal tract and thus comprise part of the gut microbial community that numbers hundreds of bacterial species, whereas others are found on plant material or in fermented foods [42]. Lactobacilli secrete bacteriocins, which kill other Gram-positive bacteria, including pathogens [41,87,88]. Furthermore, several *Lactobacillus *surface and secreted proteins have been implicated in intra-species aggregation and co-aggregation with pathogenic bacteria [88-91] and in one case have been reported to have had an impact on the expression of virulence factors of a pathogenic bacterium [92]. It has been demonstrated that probiotic Lactobacilli can modulate activation of dendritic cells [45,93-95], but the proteins mediating these effects have not yet been identified. In recent years several *Lactobacillus *genomes have been sequenced [61,62,65,66,96]. Comparative and functional analyses of these bacteria have revealed several proteins involved in colonization or adhesion [13,44,46,47,97,98]. However, focus on proteins from only a handful of *Lactobacillus *strains limits functional exploration of this genus, given that it is represented in the gut by many phylotypes [20,42,99]. Direct selection and display of the secretome at a metagenomic scale would enable bionformatic identification or functional capture of proteins with probiotic activities from numerous gut Lactobacilli and would have a potential to uncover novel probiotic strains of this genus [42].

*L. rhamnosus *HN001 is a probiotic bacterium that transiently colonizes the human gut, stabilizes the gut microflora, and enhances parameters of both innate and acquired immunity [34-36]. Our bioinformatic analysis of the *L. rhamnosus *HN001 secretome revealed a number of features in common with other probiotic bacteria, but also some distinct secretome proteins unique to *L. rhamnosus *HN001. We identified 89 ORFs encoding seven functional classes of extracellular and transmembrane proteins. *In silico *secretome analyses of the completely sequenced genomes of other Lactobacilli revealed a similar distribution of categories of predicted secretome proteins. For example, in the *L. plantarum *and *L. reuteri *secretomes the largest classes with assigned function were enzymes (30-35%) and transport proteins (10-15%), while for approximately 45% of total secretome ORFs the function of encoded proteins could not be predicted [9,100,101]. Furthermore, ORFs encoding substrate-binding domains of ABC transporters predominated among predicted *L. reuteri *transport proteins (15%) and the same was found in *L. plantarum *(14%) [65] and *L. johnsonii *(17%) [66]. A large proportion of transport proteins, enzymes and hypothetical proteins identified in these studies is consistent with our observations for *L. rhamnosus*,although compared to the other Lactobacilli, HN001 did have a somewhat higher proportion of transport proteins (25% versus 10-15%) and lower proportion of enzymes (23% versus 30-35%) These differences could be due to only partial sequencing of the HN001 secretome or may be the consequence of experimentally derived secretome data for *L. rhamnosus *HN001 versus *in silico *prediction for *L. plantarum *and *L. johnsonii*. The proportion of HN001 secretome ORFs encoding proteins that are part of the signaling system and host-microbial interaction groups (2%) was similar to observations for other species of the *Lactobacillus *genus (5%). Within this class, only one ORF, *lrh15*, encoded a protein with similarity to a histidine kinase and three ORFs (*lrh51*, *lrh35 *and *lrh62*) encoded proteins with predicted adhesion properties. Only one report has been published thus far that describes an experimentally derived secretome of a lactobacillus, *L. reuteri *DSM 20016 [71]; however, only 52 proteins were retrieved in that report. Comparison between different functional classes from *L. reuteri *DSM 20016 and *L. rhamnosus *HN001 showed similar trends; the same classes of proteins were detected and the relative proportion corresponding to each class was similar. Finally, we have identified seven unique secretome ORFs, one of which (*lrh62*) encodes a large Ala/Ser-rich surface protein unique to *L. rhamnosus *strain HN001. Considering the unique characteristics of this predicted protein, which has not yet been found in other Lactobacilli or any other bacteria, it may have a strain-specific function that distinguishes *L. rhamnosus *HN001 from other Lactobacilli, such as interacting with the host environment.

## Conclusion

Our data show that it is possible to select, with a high efficiency, the secretome of Gram-positive bacteria, by using a system consisting of a phage display phagemid vector that does not contain a signal sequence and a *gIII*-deleted helper phage. Gram-positive secretome proteins, targeted to the virion by their signal sequences, can be directly purified and functionally characterized.

Our method is sufficiently efficient to identify and display 44% of the secretome of Gram-positive bacterium *L. rhamnosus *HN001 by analyzing fewer than 500 clones from a primary library of 10^6 ^clones. When extrapolated to the metagenome scale, a comparable coverage of the meta-secretome of a complex microbial community of up to 100 species is achievable with a primary library size of 10^8 ^clones and analysis of approximately 50,000 clones.

## Materials and methods

### Bacterial strains, growth conditions and helper phage

*E. coli *strain TG1 (*supE thi-1 *Δ(*lac-proAB*) Δ(*mcrB-hsdSM*)*5 *(rK^- ^mK^-^) [F' *traD36 proAB lacI*^q^*Z*Δ*M15*]) was utilized to construct the phagemid vector pDJ01 and phage display library. *E. coli *cells were incubated in yeast extract tryptone broth (2xYT) and *E. coli *transformants in 2xYT with 20 μg ml^-1 ^chloramphenicol (Cm) at 37°C with aeration. Solid medium for growth of *E. coli *transformants also contained 1.5% (w/v) agar. *L. rhamnosus *strain HN001 was obtained from Fonterra Research Centre and was propagated in Man-Rogosa-Sharpe (MRS) broth (Oxoid, Basingstoke, Hampshire, England) at 37°C. Stocks of the helper phage VCSM13d3 with deleted *gIII *were obtained by infection of complementing *E. coli *strain K1976 (TG1 transformed with plasmid pJARA112 containing full length *gIII *under the control of phage infection-inducible promoter *psp *[49]). Helper phage VCSM13 (*gIII*^+^; Stratagene, Cedar Creek, Texas, USA) was propagated on strain TG1.

### Isolation of chromosomal DNA from *L. rhamnosus *HN001

For construction of the library, chromosomal DNA was isolated from an overnight culture of *L. rhamnosus *HN001 using a modification of the method described previously [102]. Briefly, an overnight culture was diluted 1:100 into 80 ml MRS broth and incubated overnight at 37°C. Cells were harvested by centrifugation at 5,500 × g for 10 minutes, resuspended in 80 ml of MRS broth and incubated for a further 2 h at 37°C. Cells were washed twice in 16 ml 30 mM Tris-HCl (pH 8.0), 50 mM NaCl, 5 mM EDTA and resuspended in 2 ml of the same buffer containing 25% (w/v) sucrose, 20 mg ml^-1 ^lysozyme (Sigma-Aldrich, Castle Hill, New South Wells, Austarlia) and 20 μg ml^-1 ^mutanolysin (Sigma). The suspension was incubated for 1 h at 37°C. Further lysis of the cells was accomplished by adding 2 ml 0.25 M EDTA, 800 μl 20% (w/v) SDS. After addition of SDS the suspension was carefully mixed and incubated at 65°C for 15 minutes. Next, RNase A (Roche, Basel, Switzerland) was added to a final concentration of 100 μg ml^-1 ^and the incubation was continued for 30 minutes at 37°C. Proteinase K (Roche) was added to a final concentration of 200 μg ml^-1 ^and the suspension was incubated at 65°C for 15 minutes. Finally, after phenol and chloroform extractions, the DNA was precipitated by addition of 1/10 volume 3 M sodium acetate (pH 5.2) and 2.5 volumes 95% (v/v) ethanol. The DNA was pelleted by centrifugation, washed with 70% (v/v) ethanol, air dried and resuspended in an appropriate volume of 10 mM Tris-HCl (pH 8.0).

### Construction of the new phagemid vector pDJ01

Primers pDJ01F01 (5'-GGCCCGGAAGAGCTGCAGCATGATGAAATTC-3', containing an *Ear*I site (underlined) at the 5' end) and pDJ01R01 (5'-GGGGAATTC**TCTAGA **CCCGGG**GCATGC**ATTGTCCTCTTG-3', containing, from the 5' end, *Eco*RI (first underlined sequence), *Xba*I (first bold sequence), *Sma*I (second underlined sequence) and *Sph*I (second bold sequence) restriction sites) and template pJARA144 (unpublished) were used to generate a PCR product containing the *psp *promoter followed by a ribosomal binding site and a multiple cloning site. The product was cleaved with *Ear*I and *Eco*RI and ligated into *Ear*I-*Eco*RI digested phagemid pAK100 [73]. The ligation placed the *psp *promoter, ribosomal binding site and the multiple cloning site directly upstream of a sequence encoding the peptide tag C-myc, followed by suppressible *amber *(TAG) stop codon and a coding sequence for the carboxy-terminal domain of pIII (Figure 1). The plasmid was named pDJ01.

### Construction of the phagemid displaying the SOF of *S. pyogenes*

Primers pSOF22F01 (5'-CCGCCGATGCATTGACAAATTGTAAG-3', containing an *Nsi*I site (underlined)) and pSOF22R01 (5'-CCGCCGGAATTCCTCGTTATCAAAGTG-3', containing an *Eco*RI site (underlined)) and the template, purified DNA of a λEMBL4 clone of the *sof22 *from *S. pyogenes *strain D734 (M22 serotype; The Rockefeller University Collection), were used to generate a PCR product encoding the SOF of the M22 strain, including the signal sequence but excluding the cell wall and membrane anchor sequences (963 residues). Twenty-seven cycles were used to amplify *sof22*. The thermocycling protocol started with an initial denaturation step for 2 minutes at 94°C, followed by 10 cycles of: a denaturation step (94°C for 15 s), an annealing step (59°C for 30 s) and an extension step (72°C for 2.5 minutes). A subsequent 17 cycles were carried out with the same denaturation and annealing steps but the elongation step was increased in length by 2 s in every cycle. The extension step in the final cycle was extended to seven minutes to ensure that all products were fully synthesized. The PCR product was cleaved with *Nsi*I and *Eco*RI and ligated to the *Nsi*I-*Eco*RI-cleaved vector pDJ01. This phagemid was named pSOF22.

### Production and functional assays of the SOF-displaying phagemid particles

The phagemid particles were generated by infection of 100 ml of exponentially growing cultures of TG1(pSOF22) with helper phage stocks at a multiplicity of infection of 50 phage per bacterium. Helper phages VCSM13 and VCSM13d3 were used for production of phagemid particles of the pSOF22 (named pSOF22 PP/wt and pSOF PP/d3, respectively) and VCSM13 only for production of pDJ01 (negative phagemid particle control; named pDJ01 PP/wt). VCSM13d3 helper phage was not used for the production of pDJ01 phagemid particles because of the lack of functional pIII. Infected cells were incubated for 4 h at 37°C with aeration. The host cells were pelleted by centrifugation and phagemid particles collected in the supernatant. The phagemid particles were purified by precipitation in 5% (w/v) PEG, 500 mM NaCl and resuspended in phosphate buffered saline (PBS; 125 mM NaCl, 1.5 mM KH_2_PO_4_, 8 mM Na_2_HPO_4 _and 2.5 mM KCl, pH 7.6). The phagemid particles were quantified based on the amount of phagemid DNA after disruption of the virions at 70°C in 1% (w/v) SDS as described previously [33].

The serum opacity assay was carried out by mixing 1 ml of heat-inactivated horse serum with 10^11 ^phagemid particles displaying the SOF (pSOF22 PP/wt; pSOF22/d3) or negative control (pDJ01 PP/wt) in the presence of sodium azide. The reactions were incubated at 37°C and the time course of increase of optical density over time was monitored by measuring optical density at a wavelength of 405 nm.

The fibronectin-binding assay was carried out by phage enzyme-linked immunosorbent assay (ELISA [103]). The microtiter wells (Nunc-Immuno MaxySorp™, Roskilde, Denmark) were coated with plasma fibronectin at a final concentration of 20 μg ml^-1^, 100 μl per well in PBS (pH 7.2) for 1 h at 37°C. The wells were washed once with 300 μl PBS, 0.05% Tween 20 buffer (PBST) and then blocked with 1% (w/v) bovine serum albumin (BSA) in PBS for 2 h at room temperature. The wells were then washed (three times) with 300 μl of PBST buffer. Phagemid particles (2 × 10^8^) in 100 μl of PBS were added to the wells. Negative buffer controls were TE (10 mM Tris, 1 mM, EDTA, pH 8.0), PBS, and 0.05% (w/v) BSA in PBS, and the negative phagemid particle control was pDJ01 PP/wt, generated as described above. The plates were incubated for 2 h at room temperature. The unbound phagemid particles were removed by washing with PBST (seven times). To detect bound phagemid particles, 100 μl mouse anti-pVIII (monoclonal antibody to M13, fd and f1, Progen Biotechnik, Heidelberg, Germany) at 0.1 μg ml^-1 ^in 0.1% (w/v) BSA/PBS was added and incubated for 1 h at room temperature. The wells were then washed with 300 μl PBST buffer (five times) and 100 μl secondary HRP-conjugated anti-mouse antibody was added at a dilution of 1:2,000 and incubated for 1 h at room temperature. The plate was washed seven times with PBST buffer and developed using the ImmunoPure TMB substrate kit (Pierce, Rockford, Illinois, USA). The absorbance was read at 450 nm. The phagemid particles were quantified as described above.

### Construction of the whole genome library

The library was constructed from mechanically (nebulization) sheared *L. rhamnosus *HN001 DNA and cloned into the phagemid vector pDJ01. A disposable medical nebulizer containing 1.5 ml of a buffered chromosomal DNA (approximately 20 μg) and 25% (v/v) glycerol was subjected to nitrogen gas at a pressure of 10 psi for 90 s. The fragments obtained varied in size between 0.3 and 4 kb, with the majority between 0.5 and 1.6 kb. Blunt ends were achieved by treatment with T4 DNA polymerase (Roche), Klenow fragment of DNA polymerase I (Roche) and OptiKinase™ (USB Corporation, Cleveland, Ohio, USA). To eliminate fragments below 0.3 kb, Sepharose CL-4B 200 (Sigma) size exclusion resin was used. The phagemid vector pDJ01 was digested with the restriction enzyme *Sma*I (Roche) and dephosphorylated with shrimp alkaline phosphatase (Roche). The DNA manipulations were performed according to standard methods [104].

Approximately 10 μg of the genomic fragments were ligated to 3 μg of the vector pDJ01 using T4 ligase (Roche). After phenol and chloroform extraction, the ligated DNA was ethanol-precipitated, washed with 70% (v/v) ethanol and dissolved in 25 μl H_2_O. The ligation mix was transformed into *E. coli *TG1 by electroporation (2.5 kV, 25 μF, 400 Ω) in 2-mm-gap cuvettes. The transformed cells were transferred to 50 ml of 2xYT and incubated for 1 h at 37°C with rotatory agitation. After the incubation a 2 ml aliquot was taken to determine the number of transformants by plating on 2xYT agar with 20 μg ml^-1 ^chloramphenicol. The remaining bacteria were amplified overnight at 37°C with aeration.

### Direct selection of the secretome phage display library

A 1 ml aliquot of the overnight culture containing the whole genome library was used to inoculate 25 ml of 2xYT-Cm. The exponentially growing culture (OD_600 _approximately 0.2) was infected with helper phage VCSM13d3 (multiplicity of infection = 50) for 1 h. Cells were then harvested by centrifugation at 3,200 × g for 10 minutes; the pellet was resuspended in 1 ml of 2xYT, mixed with 10 ml of soft agar (2xYT broth with 0.5% (w/v) agarose) and poured over four 2xYT-Cm plates. Both the soft agar and the plates contained molecular biology grade agarose instead of bacteriological agar. The plates were incubated overnight at 37°C, then the phagemid particles were extracted from plates by adding 5 ml of 2xYT onto each plate followed by slow rotatory agitation at room temperature for 4 h. Extracted phagemid particles were precipitated by 5% (w/v) PEG, 0.5M NaCl and resuspended in TN buffer (10 mM Tris, 150 mM NaCl, pH 7.6). To eliminate unstable (defective) phagemid particles, precipitate was treated with sarcosyl at a final concentration of 0.1% (w/v). The ssDNA released from defective phagemid particles was removed by DNase I (100 μg ml^-1^) in the presence of 5 mM MgCl_2_. DNase I was then inactivated by EDTA (20 mM). The ssDNA was then extracted from the sarcosyl-resistant virions. First the ssDNA was released from the phagemid particles by incubation at 70°C for 10 minutes in the presence of 1.2% (w/v) SDS. Further purification of the ssDNA was carried out using a plasmid mini prep kit (Roche). To amplify the secretome library from the plasmid origin of replication, *E. coli *strain TG1 was transformed with purified ssDNA.

### Sequence analysis of selected *L. rhamnosus *HN001 clones

After transformation, 491 clones were randomly selected for analysis. The phagemid DNA from these clones was purified using the 96-easy Mini-prep Kit (V-Gene Biotechnology, Hangzhou City, China). The inserts were sequenced using primer pDJ01R02 (5'-CCGGAAACGTCACCAATGAA) and BigDye^® ^Terminator v3.1 Cycle Sequencing Kit (Applied Biosystems, Foster City, California, USA) and was analyzed on a ABI3730 Genetic Analyzer (Applied Biosystems) at AWC Genome Services (Massey University). All inserts were sequenced from the 3' end, since our interest was focused on ORFs in fusion with *gIII*. Sequencing and sequence analysis was carried out in batches of 96 clones. After the sequence analysis of the first 192 clones, the clones whose sequences were detected more then five times were excluded from further sequencing using dot blot hybridization. The sequences obtained were analyzed with Vector NTI software (Invitrogen, Carlsbad, California, USA) and GLIMMER version 3.02 using a training set generated against the *L. rhamnosus *HN001 draft genome sequence, a position weight matrix representing ribosome binding sites for HN001 genes and an iterative approach, as described in the software documentation, to predict the ORFs [105]. If the 5' end of the ORF was not reached, a sequencing reaction using the forward vector-complementary primer pDJF03 (5'-ATGTTGCTGTTGATTCTTCA-3') was carried out.

SignalP 3.0 [106] and TMHMM 2.0 [107] were used for prediction of the signal sequence and transmembrane helices, respectively, using the default settings (for Gram-positive bacteria) and cut-off values [14,50]. Amino-terminally located transmembrane helices that in SignalP 3.0 analysis showed a score for the signal peptidase cleavage site (C-score) below 0.52 were considered to be amino-terminal membrane anchors. The presence of a transmembrane helix was confirmed by using the TMHMM prediction program [108]. Lipoprotein signal sequences were predicted by the LipPred server [109] using the default settings and cut-off values [53]. TATFIND 1.4 was used for prediction of Tat signal sequences [110].

All translated insert sequences were examined with BlastP [111] at the NCBI website [112] with default settings to identify similarities with other bacterial proteins. An e-value lower then e^-10 ^was used as a cut-off for notable similarity. Furthermore, conserved domains were identified in our query sequences by the Conserved Domain Architecture Retrieval Tool (CDART) engine in the course of the search; known domains being derived from either clusters of orthologous groups of proteins (COG) [113], or Pfam [114] databases.

## Abbreviations

2xYT, yeast extract tryptone broth; BSA, bovine serum albumin; Cm, chloramphenicol; ELISA, enzyme-linked immunosorbent assay; MRS, Man-Rogosa-Sharpe broth; ORF, open reading frame; PBS, phosphate buffered saline; PEG, polyethylene glycol; SOF22, serum opacity factor of *Streptococcus pyogenes*, M-type 22; ssDNA, single-stranded DNA; Tat, twin arginine translocon.

## Authors' contributions

DJ carried out 95% of the hands-on experimental work and bioinformatic analyses. The direct selection method was designed by JR and optimized by DJ. Bioinformatic analyses were carried out by DJ, MC and JR. The manuscript was written by JR and DJ. ML and MC had advisory roles in the aspects of library construction, bioinformatic analyses and input into writing of the manuscript.

## Additional data files

The following additional data are available with the online version of this paper. Additional data file 1 is a table listing all secretome ORFs, showing the signal sequences (type I and lipoprotein), the amino-terminal transmembrane anchors, internal transmembrane α-helices, annotation of the inserts and sequence accession numbers.

## Supplementary Material

Additional data file 1 A table listing all secretome ORFs, showing the signal sequences (type I and lipoprotein), the amino-terminal transmembrane anchors, internal transmembrane α-helices, annotation of the inserts and sequence accession numbers. Click here for file
